# Counteract severe heat stress by including different forms of zinc in the rabbit bucks’ diet

**DOI:** 10.1038/s41598-023-39928-3

**Published:** 2023-08-10

**Authors:** Yassmine Moemen El-Gindy, Soliman Mohamed Zahran, Mohamed Hassan Ahmed, Ahmed Mohamed Ali, Asamaa Zaid Mohamed, Sabrin Abdel-rahman Morshedy

**Affiliations:** Fish and Animal Production Department, Faculty of Agriculture (Saba Basha), Alexandria University, P.O. Box 21531, Alexandria, Egypt

**Keywords:** Physiology, Zoology

## Abstract

The harmful influences of global warming on rabbit reproduction and industry attract global attention. Zinc (Zn) is an important trace element with a wide list of functions in the male reproductive system. The aim of this study was to estimate the effects of different forms of zinc supplementation, as organic (Zn methionine), nano (nano Zn oxide) as indirect way to minimized it impact on environment, and inorganic (Zn sulphate) on physiological parameters, semen quality, anti-oxidative status, hormonal profiles of male rabbits subjected to server heat stress. Thirty-six V-line bucks (6–7 months old, 2842.29 ± 34.46 g weight) were randomly distributed to 4 groups, bucks in 1st group (control group, Con) fed basal diet without Zn supplementation, the 2nd, 3rd and 4th groups fed basial diet with 30 mg/kg of zinc methionine (Zn-Met), nano zinc oxide (ZnO-NPs), and zinc sulphate (Zn-S), respectively for a period of 10 weeks suffered from severe heat stress of environmental ambient temperature (over 32 of temperature humidity index, THI). Semen samples were collected and evaluated for volume, pH, motility, concentration, viability, initial fructose, and seminal plasma antioxidant concentration. Liver, kidney function, hormonal and antioxidant profile were measured in blood serum. The results revealed that, compared to control, all forms of Zn supplementation used significantly improved kidney function (creatinine), serum antioxidant (SOD and CAT), physiological parameters, especially on 1st month of the experiment, and seminal plasma antioxidant (SOD and CAT) of heat stressed bucks. Likewise, semen quality in terms of sperm concentration, sperm viability, and initial fructose enhanced significantly (*P* ≤ 0.05) by ZnO-NPs supplementation. Zinc methionine supplementation significantly improved liver function and decreased seminal plasma TBARs. Treatments with Zn-Met and ZnO-NPs increased seminal TAC and blood testosterone levels with reduced blood cortisol levels compared to other groups. Severe heat stress could be counteract by inclusion Zn with studied forms Zn-Met and ZnO-NPs at recommended dose (30 mg/kg diet) to improve semen quality and antioxidant status.

## Introduction

Recently, global warming and heat stress represent the main challenges that contribute to the significant reduction in rabbit semen quality, leading to a significant decrease in sexual behavior, hormonal imbalances, an increase in abnormal sperm; a drop in sperm production, and finally a decrease in fertilization capacity and fertility^1^, especially with the highly sensitively to heat stress due to lack of sweat glands, except the ear^1^. Moreover, many blood biochemicals dis-balances in male rabbit were occurred by exposure to thermal stress^2^. Sperm are rich in polyunsaturated fatty acids with low levels within the cytoplasm^3^. Heat stress caused a higher level of inflammatory responses in the blood, resulting in a decrease in immunological function and a potential for apoptosis spermatozoa^4^. In addition, thermal stress caused an increase in oxidative stress, which in turn increased tissue damage by accelerating lipid peroxidation^2^ and significantly weakening antioxidant defense^5^, with lipid peroxidation followed in cellular damage^6^.

Several methods have been used to prevent the negative impacts of heat stress in buck rabbits, including phytogenic, minerals, and vitamins^7^. Many microelements are successfully used to reduce the damaging effects or avoid harmful impacts of heat stress^1^. Zinc is one of many series components that control the oxidative stress of cells^8^. In addition, zinc is the second richest trace element after iron, which plays an important role in the male reproductive process because of their high activity at the molecular level^9,10^, and involved in various enzyme activities and hormone functions^11^. Zinc is an effective antioxidant owing key role to catalytic, coactive, or structural roles in different cofactor including more than 300 enzymes^12^ such as SOD; more than 2000 transcription factors^13^, involved in the biosynthesis of DNA and cell division^14^, gene expression^15^, and immune protection^16^. Overall, zinc acts as an antioxidant stress agent by inhibition of oxidation of macromolecules such as DNA and proteins, as well as the inhibition of inflammatory response, subsequently downregulation of ROS production^17^. Inorganic zinc bioavailability in animals is low and large amounts of zinc are excreted into faeces, resulting in environmental pollution and waste of zinc resources. Nano-zinc can be used at lower doses and indirectly prevents environmental contamination^18^. The nano-zinc (particle size is below 100 nm) is more effective, available, and absorbed easier^19^, while it bypasses through the stomach wall and into body cells more quickly than ordinary minerals with larger particle size^20^.

Therefore, the objective of the present study was to determine whether the use of various zinc sources, such as zinc methionine, nano zinc oxide and zinc sulphate, under heat stress conditions could improve the semen of the quality of the rabbit bucks, their hormonal status, and their antioxidant status.

## Material and method

### Location and ethics statement

The study was carried out at the Department of Animal and Fish Production, Faculty of Agriculture (Saba- Basha), Alexandria University, Alexandria, Egypt (31° 12′ 20.7108′′ N, 29° 55′ 28.2936′′ E). All experimental procedures and handling of buck rabbits were approved by the Alexandria University Institutional Animal Care and Use Committee (Approval Number: 03/19/03/18/1/15), during the summer season (July to September 2021). The experiment was carried out according to relevant guidelines and regulations. Our animal research reports follow the recommendations of the ARRIVE Guidelines.

### Zinc source, animal, and diets

Thirty-six fertile V-line male rabbits, 6–7 months old with body weight (2842.29 ± 34.46 g), were used. Live body weights of rabbit bucks were recorded at the beginning of the experiment and at the end. Body weight change was calculated. The bucks were individually caged in wire batteries with a normal daylight (16–17 h). All rabbits were adapted in the experimental rabbit shed for 2 weeks. The rabbits were randomly distributed into four groups (n = 9 bucks per group) as follows: the first group (control group) fed a base diet without Zn supplementation, the second, third and fourth groups fed a base diet with 30 mg/kg of zinc methionine (Zn-Met, Mintrix^®^ Zn was purchased from United BioMed for feed additives, Cairo, Egypt), nano zinc oxide (ZnO-NP, size of < 100 nm; surface area m^2^ g^−1^, purity > 97 obtained by Sigma-Aldrich, Saint Louis, USA) and zinc sulphate (ZnS provided by El- Gomhoria Co. for Chemicals and Pharmaceuticals, Alexandria, Egypt), respectively according to recommendation doses of^21,22^ for ZnO NPs and close to recommendation doses of^23^ for Zn-Met and ZnS**.** The experiment was carried out according to relevant guidelines and regulations. The experiment lasted 10 weeks. Feed and fresh clean water were provided ad libitum. The basic diet was formulated as (16% yellow corn grain, 7% barley, 19% wheat bran, 20% soybean, 24% clover hay, 10% wheat straw, 2% beet molasses, 0.3% premix, 0.2% calcium carbonate, 0.8% dicalcium phosphate, 0.5% salt, 0.15% l-lysine, 0.05% methionine DL) and the calculated chemical composition was (88.71% dry matter, 17.21% crude protein, 14.81% crude fiber, 2.47% fat, 8.25% ash) according to the recommendation of^24^.

### Microenvironment data

Throughout the experimental period (10 weeks), ambient temperature (°C) and relative humidity (%) were recorded daily at midday using a Mercury-room thermometer and hygrometer, respectively. the weekly temperature-humidity index (THI) was determined by the equation defined by^25^**.** The mean values of THI were classified according to^26^**.**

### Physiological parameters

The rectal temperature (RT) and the respiration rate (RR) were recorded twice (4th and 8th weeks of the experiment) at 12:00–2:00 PM. A clinical digital thermometer and stopwatch were used to measure RT by inserting the thermometer 2 cm into the rectum for 2 min, inclined toward the wall of the rectum. Visual counting of nasal movement per 1 min without disturbing the rabbits by using a hand counter to determine the RR.

### Semen collection and evaluation

The semen samples of each rabbit buck were collected weekly in the morning (8 am) for 10 weeks using an artificial vagina by exposing a mature female rabbit to the bucks as a teaser during collecting. After collection, the ejaculate volume without gel was recorded in graduated collecting tubes to the nearest 0.01 ml (excluding the gel plug). Semen pH was directly measured by using pH paper strips (range: 0–14, resolution: 1.0 pH unit; Sigma-AldrichVR). Sperm mass activity was determined manually according to^27^, giving an arbitrary score of 0 to 3. The individual progressive motility was examined by diluted semen with normal saline (1:100) and transferred to a warm glass slide (37 °C) covered with a cover slip and examined under a light microscope (400× magnification) on a scale of 0 to 100% according to^28^. Sperm concentration (*10^6^/mm^3^) was evaluated in duplicate using a Neubauer hemocytometer after dilution with distilled water (1:200). Sperm viability was determined using semen smears mixed with Eosin-Nigrosin stain and viable and non-viable spermatozoa were determined by counting 200 sperm under a light microscope^29^. The initial fructose concentration of the collected semen was determined by a spectrophotometer using commercial kits (Biodiagnostic, Egypt) according to the manufacturer's instructions. At the 10th week of the experiment, the seminal plasma of 4 bucks of each treatment was separated from the mass of the sperm cells by centrifugation at 700*g* for 15 min. The supernatant (seminal plasma) was collected and kept at − 20 °C until analysis. Seminal plasma SOD, CAT, TBARs, and TAC were verified by colorimetric techniques using commercial kits (purchased from Bio-diagnostic, Giza, Egypt), according to the manufacturers’ instructions.

### Blood biochemical

At the end of 10th week of the experiment period, blood samples were collected morning before adding feed, from the margin ear of bucks after disinfecting with a sanitizer solution in sterilized tube. Then the blood samples were centrifuged at 700*g* for 10 min to obtain serum. The serum obtained was transferred to sterile Eppendorf^®^ tubes and stored at − 20 °C. Serum samples were analyzed for AST and ALT according to^30^, urea^31^, creatinine^32,33^, SOD^34^, CAT^35,36^, TBARS^37^, and TAC^38^. These analyses were performed using commercial kits (acquired from Bio-diagnostic, Giza, Egypt).

The cortisol (MBS2005085) and testosterone (MBS704954) enzyme-linked immunosorbent assay (ELISA) MyBioSource kits (sunny Southern California, San Diego, USA) were employed in quantifying the concentrations of cortisol and testosterone, respectively, following the manufacturer’s protocols.

### Statistical analyses

The experimental data were subjected to a one-way analysis of variance analyzed (ANOVA) via the GLM procedure using Statistical Analysis Software (IBM SPSS Statistics for Windows, Version 20, Chicago, IL, USA). Each buck rabbit is an experimental unit that provides an independent measurement. All percentage data were arc-sine transformed prior to the approximate normal distribution. The Tukey test was used to compare the differences between the means when significant F values were observed at the levels of *p* < 0.05, according to the following statistical model: One-way model: YiK = μ + Xi + eik where: YiK = the response variable; μ = the overall mean; Xi = the fixed effect of treatment (control, 30 mg Zn MET, 30 mg Naon ZnO, and 30 mg ZnSo_4_); eik = the residual error. Figures were fitted by the SigmaPlot (software 14.0 Systal software Inc).

### Ethics approval and consent to participate

All experimental procedures and handling of buck rabbits were approved by the Alexandria University Institutional Animal Care and Use Committee.

## Results

### Microclimate and physiological parameters

**Figure 1:**
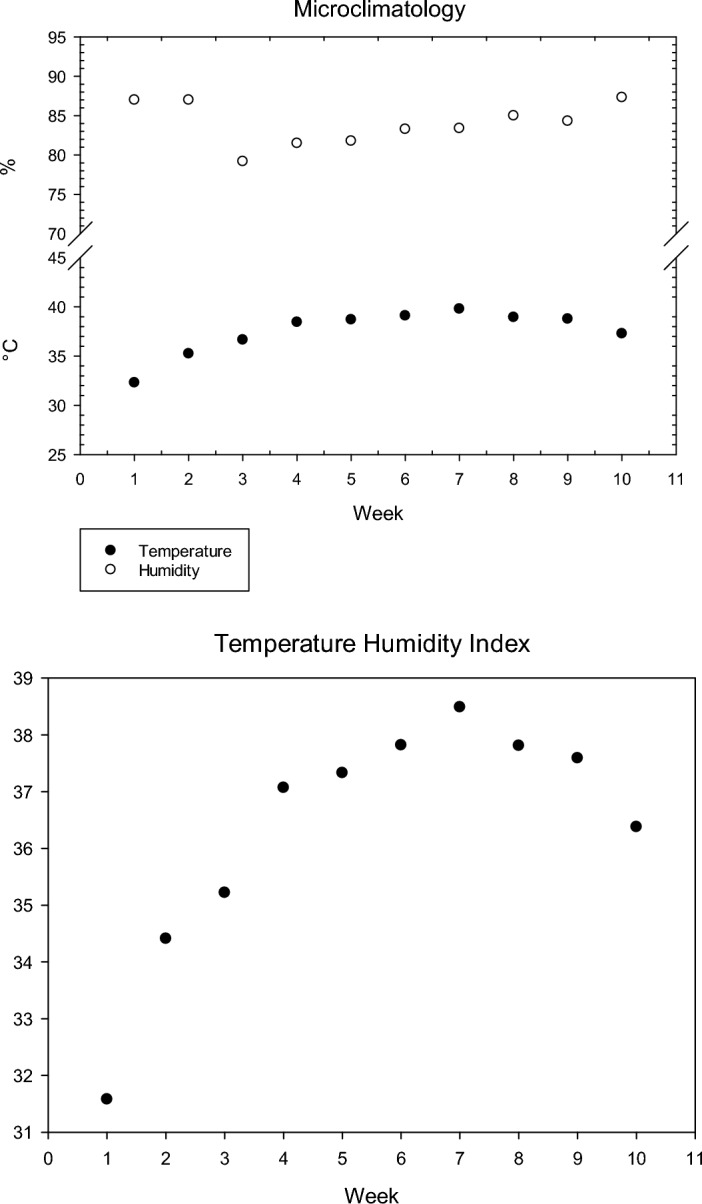
Microclimate date of the experimental period (temperature, humidity, and temperature humidity index, THI).

**Figure 2:**
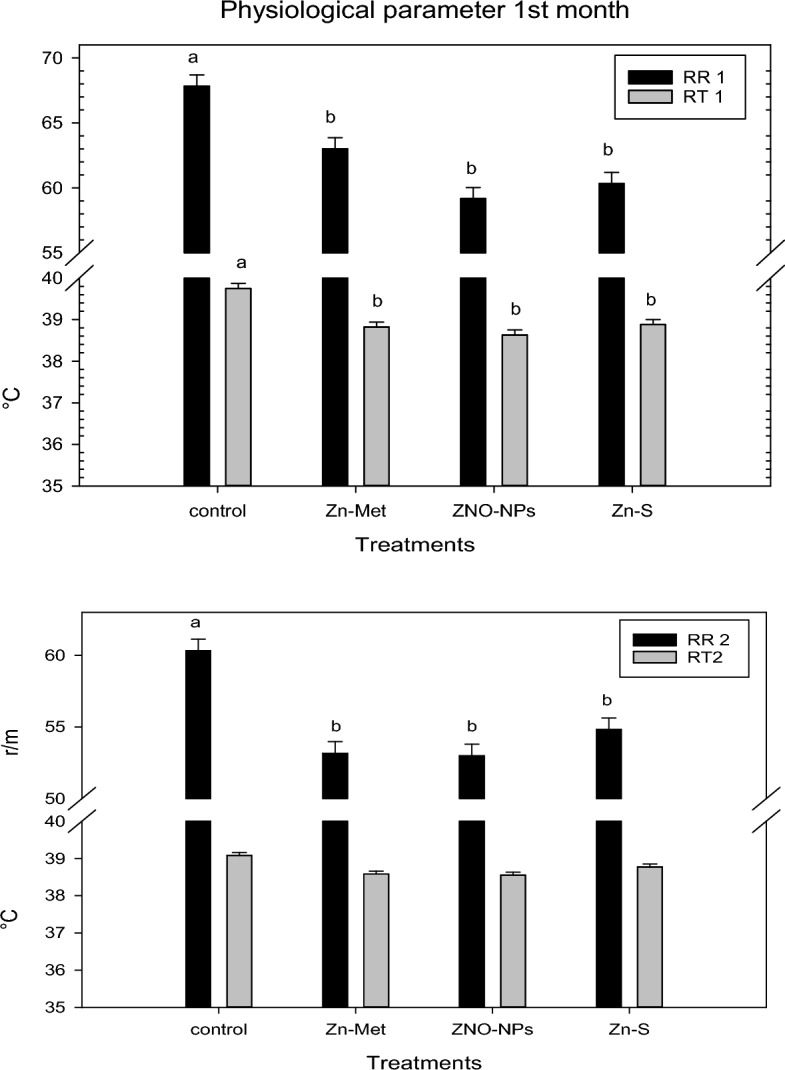
Effect of the different zinc sources on physiological parameters, respiration rate (RR) and rectum temperature (RT), of heat stressed V-line buck rabbits during 1st half and 2nd half of the experiment. Zn-Met = zinc methionine; ZnO-NPs = zinc oxide nanoparticales; Zn-S = zinc sulphate.

Figure 1 shows the weekly ambient temperature, humidity, and THI during the experiment period. The weekly temperature humidity index under the experimental conditions ranged from 31.58 to 38.49. The recorded THI values clearly showed that the bucks suffered severe heat stress. Physiological parameters (RR and RT) during 1st and 2nd months of the experimental period are presented in Fig. 2. The result revealed that all sources of Zn supplementation in this study significantly succeeded in decreasing both RR and RT values of heat stressed bucks on the first month and RR only on the second month. From Fig. 2 it can be shown that the inclusion of zinc in heat-stressed buck rations still has an effect on RT but the effect is not significant.

### Body weight and semen quality

The data in Table 1 indicated that the initial and final body weight of heat stressed bucks did not differ in all experimental groups. While the change of body weight due to zinc treatments was significant compared to the control group Table 2 shows that the most semen parameters were significantly improved by zinc supplementation except semen pH, mass motility and individual motility.. The highest semen volume was collected from the ZnS group. While both ZnO NPs and ZnS significantly recorded the highest sperm concentration compared to other treatments. All zinc treatments significantly enhanced the percentage of viability of the sperm. The seminal plasma initial fructose level (*P* < 0.05) was increased by inclusion zinc with different forms in heat stressed bucks’ rations compared to the control group and this effect was significant with zinc Met and ZnO NPs only.

**Table 1:** Effect of the different zinc sources on body weight of heat stressed V-line buck rabbits.

Parameters	Zinc sources				SEM	*P* value
	Control	Zn-Met	ZnO-NPs	Zn-S		
Initial body weight (g)	2885.83	2877.50	2800.00	2805.83	34.46	0.751
Final body weight (g)	3634.17	3860.83	3885.00	3846.67	36.86	0.046
Boy weight change (g)	748.33^b^	983.33^a^	1085.00^a^	1040.83^a^	31.37	0.001

Means in row with different superscript letters (a–c) are significantly different (*p* ≤ 0.05).

Zn-Met = zinc methionine; ZnO-NPs = zinc oxide nanoparticales; Zn-S = zinc sulphate.

**Table 2:** Effect of the different zinc sources on semen quality of heat stressed V-line buck rabbits.

Parameters	Zinc sources				SEM	P Value
	Control	Zn-Met	ZnO-NPs	Zn–S		
Semen volume (ml)	0.36^b^	0.41^ab^	0.44^ab^	0.48^a^	0.015	0.042
Semen pH	7.10	7.17	6.95	7.40	0.093	0.464
Mass motility (1–3)	1.38	1.50	1.38	1.50	0.044	0.584
Individual motility (%)	87.99	89.23	88.53	89.63	0.577	0.769
Sperm concentration (*10^6^/ml)	157.90^b^	170.88^ab^	173.60^a^	177.90^a^	2.209	0.010
Sperm viability (%)	71.63^b^	73.69^a^	73.35^a^	73.21^a^	0.287	0.049
Fructose (mg/dl)	213.03^c^	224.54^ab^	229.54^a^	221.12^bc^	1.712	0.001

Means in row with different superscript letters (a-c) are significantly different (*p* ≤ 0.05).

Zn-Met = zinc methionine; ZnO-NPs = zinc oxide nanoparticales; Zn-S = zinc sulphate.

**Figure 3:**
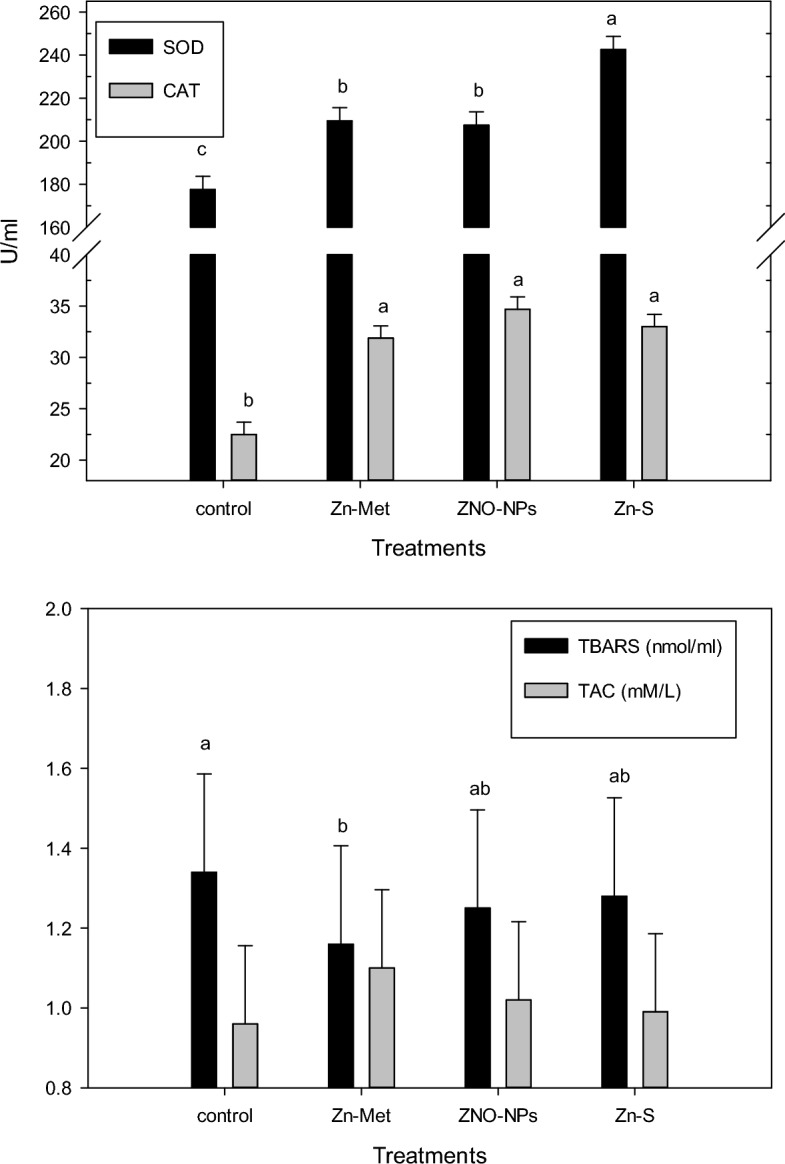
Effect of the different zinc sources on seminal plasma antioxidant status of heat stressed V-line buck rabbits. Zn-Met = zinc methionine; ZnO-NPs = zinc oxide nanoparticales; Zn-S = zinc sulphate.

The antioxidant status of seminal plasma of heat stressed bucks such of SOD and CAT levels (Fig. 3) were significantly improved by zinc treatment with different sources. As well, the TBAR concentration in seminal plasma showed a decrease in all zinc supplementation groups compared to the control group and this decrease was significant with Zn Met.

### Biochemical and hormonal analysis

**Figure 4:**
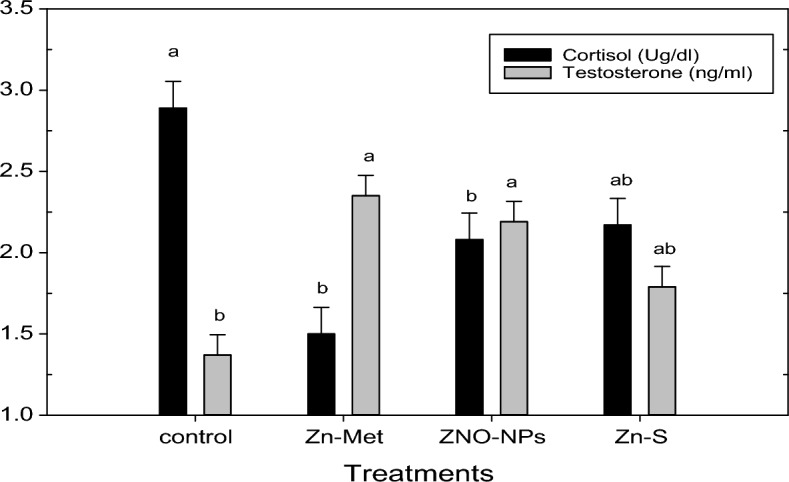
Effect of the different zinc sources on blood hormones of heat stressed V-line buck rabbits. Zn-Met = zinc methionine; ZnO-NPs = zinc oxide nanoparticales; Zn-S = zinc sulphate.

As described in Table 3, liver functions (serum concentrations of AST and ALT) were decreased significantly with Zn-Met supplementation compared to the control group, while the other Zn treatments did not differ than the control group. All zinc treatments improved kidney function as reflected in decreasing creatinine level. From Table 3, blood antioxidant status, whereas, SOD, CAT and TAC were improved and TBARS was decreased by adding Zn from different sources to heat stressed bucks rations and this effect was significant with SOD and TAC. Furthermore, the results of our study in Fig. 4 showed that all zinc treatments decreased serum cortisol levels with elevated serum testosterone concentrations of heat stressed bucks.

**Table 3:** Effect of the different zinc sources on liver and kidney function blood biochemical of heat stressed V-line buck rabbits.

Parameters	Zinc sources				SEM	P Value
	Control	Zn-Met	ZnO-NPs	Zn-S		
Liver function						
AST (U/ml)	52.29^a^	43.12^b^	44.70 ^ab^	48.45^ab^	1.190	0.019
ALT (U/ml)	54.55^a^	45.80^b^	51.46^ab^	50.90^ab^	1.059	0.018
Kidney function						
Urea (mg/dl)	25.56	26.52	25.16	25.74	0.616	0.899
Creatinine (mg/ml)	1.46^a^	0.95^b^	0.96^b^	1.00^b^	0.049	0.001
Antioxidant status						
SOD (U/ml)	45.24^b^	56.23^a^	53.24^a^	52.75^a^	1.042	0.001
Catalase (U/ml)	145.73	156.88	153.12	150.00	1.637	0.090
TBARS (NMPOL/ML)	0.90	0.61	0.72	0.74	0.081	0.678
TAC (mM/L)	1.54^b^	1.88^a^	1.84^a^	1.78^ab^	0.045	0.019

Means in row with different superscript letters (a–b) are significantly different (*p* ≤ 0.05).

Zn-Met = zinc methionine; ZnO-NPs = zinc oxide nanoparticales; Zn-S = zinc sulphate.

## Discussion

Temperature humidity index (THI) is used to evaluate the degree of heat stress by the combined both ambient temperature and relative humidity estimate the severity of heat stress. This index is widely used in hot and humid areas around the world to assess the impact of heat stress^39^. The weekly THI values clearly indicated that the buck rabbits suffered severe thermal stress. Zinc is an effective antioxidant and plays a vital physiological role for animal health, synthesis of more than 300 enzymes involved in metabolism process^40^, synthesis proteins, immune system activation, bone development^41^, DNA synthesis, cell division, gene expression, and biological functions^42^. In addition to reducing the negative impact of both cold and heat stressors^43^. Zinc methionine is organic forms of Zn are generally bound to methionine amino-acid complex, it may be better absorption and more available to animal by interaction with other elements, especially Cu, Fe, and Ca^44^. Nano-zinc oxide is smaller than 100 nm causes increasing absorption rate and availability^40^, and is more biologically effective^45^ by bypassing conventional physiological ways of nutrient distribution, and transport across tissue and cell membranes, as well as protecting compounds against destruction before reaching their targets^19^, and reaching target cells more quickly than ordinary minerals with larger particle size^20^. At the same time, using nano-Zn oxide is useful to avoid excessive supplementation with Zn in animal diets and lowest the accumulation of Zn in soils after intensive animal farming^46^, thus reducing the potential risks for environmental pollution^18,47^.

Physiological parameters such as respiration rate and rectum temperature are considered direct indexes of climatic stress^48^ and accordingly measure the degree of discomfort/comfort or adaptability of bucks. Our result revealed that all sources Zn supplementation significantly decrease RR and RT values of heat stressed bucks on 1st month and RR only on 2nd month. The positive impact of zinc on physiological parameter of heat stressed buck may be due to that zinc reduced stress on the rabbits by enhancing the bioavailability of other minerals^49^. The insignificant RT values in the 2nd month of all zinc treatments may be due to the development of metabolic mechanisms of all experimental bucks to adapt to heat stress to a tolerable level.

The initial and final body weights of the heat-stressed bucks did not differ in all experimental groups. Although the body weight change of all zinc treatments was significantly greater compared to the control bucks, that cleared that all zinc treatments alleviated the negative effect of heat stress.

Zinc sulphate treatment significantly improved semen volume, sperm concentration and sperm viability; a similar improvement was obtained by^50,51^ who concluded a significant increase in semen volume, total live sperm concentration and sperm mobility due to zinc sulphate dietary supplementation in heat-stressed rabbit males. Increased semen volume of Zn-s treatment may contribute to increased prostatic fluid^9^. Furthermore, nanozinc significantly increased each of sperm concentration, sperm viability, and initial fructose compared to the control group could be due to improvement in buck antioxidant status^52^. Similar results were found by^45^ who reported that nanoparticles have biological effects that improve male fertility by enhancing sperm quality. Also,^53^ observed that supplemented young rams’ diet with 50 mg/kg or 100 mg/kg nano zinc practical improved epididymal semen quality. Increasing the percentage of sperm viability of nanozinc bucks may be due to improvement the functionality of sperm plasma membranes without any significant effect on motility parameters^54^. Furthermore, the improvement in sperm concentration of bucks fed a diet supplemented with nano-zinc could be due to increased antioxidant enzymes activity of testicular tissue^55^ or seminal plasma in the current study that protects sperm against oxidative stress. In addition, Zn-Met significantly enhanced the viability sperm percentage and initial fructose in the present findings.

Additionally, the membrane of rabbit sperm cells is rich in polyunsaturated fatty acids that make sperm susceptible to lipid peroxidation^56^. Other semen characteristics such as pH and motility (mass and individual) were not differ among experimental groups. Our results agree with those of^57^ and^58^ who reported that supplemental organic and non-organic zinc improved quantitative and qualitative characteristics of semen reproductive efficiency^59^ and fertility by lower blood kidney parameters that decrease ROS production and increased progressive motility, acrosome reaction and capacitation^60^. Viable sperm percentage did not effect by all zinc treatments in the current results, this observation has a harmoniousness with previous studies of^9,61^. In general, zinc affects reproductive characteristics by stimulating and protecting the germinal epithelium of seminiferous tubules and promoting the production and secretion of testosterone^62^, as shown in the present findings, and therefore influences spermatogenesis^63^. Besides, the positive result of all zinc treatments on sperm concentration and spermatogenesis may be due to the production of sperm necessitates extensive cell division by influencing mitotic and meiotic cell divisions, synthesis of DNA and RNA by improving the activity of DNA and RNA polymerase (Zn-containing enzymes)^64^. Increasing initial fructose in all zinc treated bucks as compared to the control bucks may be attributed to the effect of treatments on fructogenesis in seminal vesicles of heat stressed rabbits.

Antioxidant status such as SOD and CAT levels of the seminal plasma of heat-stressed bucks in the present findings were significantly improved by different sources of zinc treatments. Although zinc methionine supplementation was more effective in reducing the seminal plasma TBAR concentration compared to other groups. These results agreed with those presented by^65^ who reported that Zn supplementation had a beneficial effect on the anti-oxidative status of seminal plasma, which may provide better protection to spermatozoa from oxidative damage, and also improve the concentration of testosterone as is shown in the current study which may play a role in the production of good quality semen.

Liver functions were significantly improved as AST and ALT concentrations were reduced in Zn-Met group compared to the control group with no significant effect of other zinc treatments. The positive effect of Zn treatments on semen quality may be due to the revealed negative relationship between liver enzymes such as ALT and GGT and sperm concentration, total testosterone and LH due to the formation of pro-oxidant species formation^66^. Our results were in agreement with^67,68^ who found that supplementation with ZnO-NP in rabbit diets significantly reduced serum levels of AST and ALT activities compared to the control group. All zinc treatments improved kidney function as reflected in the decrease in creatinine values. The current results were agreed with^69,70^ that found the diets supplemented with 50 and 100 mg/kg of Zn-Met significantly decreased blood creatinine concentration compared to the control group. On the other hand, the result disagreed with^71^ who reported that plasma creatinine concentration did not change in rabbit fed diet supplemented with Zn.

Heat stress decreases antioxidant status, which is one of the most important physiological changes in response to high ambient temperature in animals that increases oxidative stress and immune suppression^72^. Dietary supplementation of different Zn sources in the current study improved the serum antioxidant status (SOD and TAC levels) with no significant effect between ZnO NPs and control groups. The improvement in blood antioxidant status may be due to zinc being considered an effective antioxidant by having catalytic, coactive, or structural functions in different enzymes such as SOD, which controls many physiological processes such as metabolism and immune function^73^, or by inhibiting the oxidation of macromolecules such as DNA and proteins^17^ or by maintaining the activities of radical scavenging enzymes^40^. The improvement in blood antioxidant status may be associated to the enhancing in seminal antioxidant status as mentioned earlier in our study, which illustrated that zinc prevents the oxidative damage to the sperm cells and thereby improve the fertility^11^. From Fig. 3 TBARs seminal plasma was affected by Zn supplementation, whereas, TBARs was decreased in Zn treatments compared to the control group but this effect was nonsignificant. Our results disagree with the findings of^74^ who showed that plasma TBARs and plasma lipid peroxides concentration was reduced significantly by zinc supplementation in rabbit diets.

Animals that exposed to ecological stressors such as extremes of temperatures, neurons in the CNS are stimulated, that activated of the hypothalamic pituitary adrenal axis leading to stimulate the cortex of the adrenal gland to produce and release corticosteroids (cortisol)^75^. However, zinc had an effect on the severe heat stress by decreasing cortisol levels which observed in the current study, whereas all zinc treatments declined serum cortisol levels. Testosterone as a sex hormone has a positive impact on semen quality and reproductive physiology^76^. In the present findings, all zinc forms led to elevated serum testosterone concentration of heat stressed bucks, which reflected the improvement in semen characteristics which was in consistency with previous studies. Many studies supported our results on improving blood testosterone concentration^61^**,** which helps in building protein, and is necessary for sexual desire and erections^77^.

## Conclusions

Current data revealed a significant positive improvement in body weight change, blood kidney function (creatinine), serum antioxidant (SOD and CAT), physiological parameters, especially in the 1^st^ month of experiment, and seminal plasma antioxidant (SOD and CAT) of heat stressed bucks with the three forms of dietary Zn supplementation. Furthermore, semen quality in terms of sperm concentration, viability, and initial fructose improved significantly with ZnO-NPs supplementation. Zinc methionine supplementation significantly improved liver function and reduced seminal plasma TBARs. Both ZnO-NPs and Zn-Met improved seminal TAC and blood testosterone with reduced blood cortisol levels. Therefore, it concluded that the recommended forms of Zn in the dose of 30 mg/kg diet are zinc methionine and ZnO-NPs, rather than ZnS during the severe heat stress conditions to improve semen quality, and serum and seminal antioxidant status of buck rabbits.

## Data Availability

The data used to support our study’s findings are included in the article, and data coding is available from the corresponding author upon reasonable request.

## Abbreviations

ANOVA: A one-way analysis of variance analyzed
CAT: Catalase
CNS: Central nerves system
DNA: Deoxyribonucleic acid
ELISA: Enzyme-linked immunosorbent assay
AST: Aspartate transaminase
ALT: Alanine transaminase
pH: Potential of hydrogen
RR: Respiration rate
RT: Rectal temperature
SOD: Superoxide dismutase
TBARs: Thiobarbituric acid reactive substances
THI: Temperature humidity index
Zn: Zinc
Zn-Met: Zinc methionine
ZnO-NPs: Nano zinc oxide
ZnS: Zinc sulphate

## References

[1] Sheiha AM (2020). Effects of dietary biological or chemical-synthesized nano-selenium supplementation on growing rabbits exposed to thermal stress. Animals.

[2] Hosny NS, Hashem NM, Morsy AS, Abo-Elezz ZR (2020). Effects of organic selenium on the physiological response, blood metabolites, redox status, semen quality, and fertility of rabbit bucks kept under natural heat stress conditions. Front. Vet. Sci..

[3] El-Gindy YM (2021). Improvement in quality and storage ability of rabbit semen by using black or thyme seed as dietary supplementation. J. Anim. Physiol. Anim. Nutr. (Berl.).

[4] Chaidanya K (2020). Impact of heat stress, nutritional stress and combined (heat and nutritional) stresses on rumen associated fermentation characteristics, histopathology and HSP70 gene expression in goats. J. Anim. Behav. Biometeorol..

[5] Rezaei N (2018). Effects of l-Carnitine on the follicle- stimulating hormone, luteinizing hormone, testosterone, and testicular tissue oxidative stress levels in streptozotocin-induced diabetic rats. J. Evid.-Based Integr. Med..

[6] Gaschler MM, Stockwell BR (2017). Lipid peroxidation in cell death. Biochem. Biophys. Res. Commun..

[7] Farghly MFA, Alagawany M, Abd El-Hack ME (2018). Feeding time can alleviate negative effects of heat stress on performance, meat quality and health status of turkey. Br. Poult. Sci..

[8] Marklund S (1980). Distribution of CuZn superoxide dismutase and Mn superoxide dismutase in human tissues and extracellular fluids. Acta Physiol. Scand. Suppl..

[9] Zhao J (2016). Zinc levels in seminal plasma and their correlation with male infertility: A systematic review and meta-analysis. Sci. Rep..

[10] Oliveira, C. E. A., Badu, C. A., Ferreira, W. M., Kamwa, E. B. & Lana, A. M. Q. Effects of dietary zinc supplementation on spermatic characteristics of rabbit breeders. in *8th World Rabbit Congress, Mexico* 7–10 (2004).

[11] Hemalatha K (2018). Effect of dietary supplementation of organic zinc and copper on in vitro semen fertility in goat. Small Rumin. Res..

[12] Abd El-Hack ME (2018). Effect of dietary supplementation of organic zinc on laying performance, egg quality and some biochemical parameters of laying hens. J. Anim. Physiol. Anim. Nutr. (Berl.).

[13] Xu X (2017). Effects of chromium methionine supplementation with different sources of zinc on growth performance, carcass traits, meat quality, serum metabolites, endocrine parameters, and the antioxidant status in growing-finishing pigs. Biol. Trace Elem. Res..

[14] Mateos, G. G., Rebollar, P. G. & de Blas, C. Minerals, vitamins and additives, in *Nutrition of the Rabbit* 119–150 (CABI Wallingford UK, 2010).

[15] Macpherson LJ (2005). The pungency of garlic: Activation of TRPA1 and TRPV1 in response to allicin. Curr. Biol..

[16] Yan JY, Zhang GW, Zhang C, Tang L, Kuang SY (2017). Effect of dietary organic zinc sources on growth performance, incidence of diarrhoea, serum and tissue zinc concentrations, and intestinal morphology in growing rabbits. World Rabbit Sci..

[17] Prasad AS, Bao B (2019). Molecular mechanisms of zinc as a pro-antioxidant mediator: clinical therapeutic implications. Antioxidants.

[18] Swain PS, Rao SBN, Rajendran D, Dominic G, Selvaraju S (2016). Nano zinc, an alternative to conventional zinc as animal feed supplement: A review. Anim. Nutr..

[19] Hett A (2004). Nanotechnology: Small Matter, Many Unknowns.

[20] Bunglavan SJ, Garg AK, Dass RS, Shrivastava S (2014). Use of nanoparticles as feed additives to improve digestion and absorption in livestock. Livest. Res. Int.

[21] Hassan FAM, Mahmoud R, El-Araby IE (2017). Growth performance, serum biochemical, economic evaluation and IL6 gene expression in growing rabbits fed diets supplemented with zinc nanoparticles. Zagazig Vet. J..

[22] Qureshi AA, Abuirmeileh N, Din ZZ, Elson CE, Burger WC (1983). Inhibition of cholesterol and fatty acid biosynthesis in liver enzymes and chicken hepatocytes by polar fractions of garlic. Lipids.

[23] Luis-Chincoya H, Herrera-Haro JG, Pro-Martínez A, Santacruz-Varela A, Jerez-Salas MP (2021). Effect of source and concentration of zinc on growth performance, meat quality and mineral retention in New Zealand rabbits. World Rabbit Sci..

[24] National Research Council. Nutrient requirements of rabbits. (1977).

[25] Marai IFM, Habeeb AAM, Gad AE (2002). Rabbits’ productive, reproductive and physiological performance traits as affected by heat stress: A review. Livest. Prod. Sci..

[26] LPHSI, A. *Livestock and Poultry Heat Stress Agriculture engineering technology guide*. (Clemson University, Clemson, SC. USA, 1990).

[27] Moule GB (1965). Field Investigations with Sheep. A Manual of Techniques.

[28] Amann RP, Hammerstedt RH (1980). Validation of a system for computerized measurements of spermatozoal velocity and percentage of motile sperm. Biol. Reprod..

[29] Shaffer HE, Almquist J (1948). Vital staining of bovine spermatozoa with an Eosin-Aniline blue staining mixture (abstract). J. Dairy Sci..

[30] Reitman S, Frankel S (1957). A colorimetric method for the determination of serum glutamic oxalacetic and glutamic pyruvic transaminases. Am. J. Clin. Pathol..

[31] Fawcett J, Scott J (1960). A rapid and precise method for the determination of urea. J. Clin. Pathol..

[32] Bartels H, Böhmer M, Heierli C (1972). Serum creatinine determination without protein precipitation. Clin. Chim. Acta..

[33] Larsen K (1972). Creatinine assay by a reaction-kinetic principle. Clin. Chim. Acta..

[34] Nishikimi M, AppajiRao N, Yagi K (1972). The occurrence of superoxide anion in the reaction of reduced phenazine methosulfate and molecular oxygen. Biochem. Biophys. Res. Commun..

[35] Aebi H (1984). Catalase in vitro. Methods Enzymol..

[36] Fossati P, Prencipe L, Berti G (1980). Use of 3,5-dichloro-2-hydroxybenzenesulfonic acid/4-aminophenazone chromogenic system in direct enzymic assay of uric acid in serum and urine. Clin. Chem..

[37] Tappel AL, Zalkin H (1959). Inhibition of lipide peroxidation in mitochondria by vitamin E. Arch. Biochem. Biophys..

[38] Koracevic D, Koracevic G, Djordjevic V, Andrejevic S, Cosic V (2001). Method for the measurement of antioxidant activity in human fluids. J. Clin. Pathol..

[39] Asemota OD, Aduba P, Bello-Onaghise G, Orheruata AM (2017). Effect of temperature-humidity index (THI) on the performance of rabbits (*Oryctolaguscuniculus*) in the humid tropics. Arch. Zootec..

[40] Sallam AE, Mansour AT, Alsaqufi AS, Salem ME-S, El-Feky MMM (2020). Growth performance, anti-oxidative status, innate immunity, and ammonia stress resistance of Siganus rivulatus fed diet supplemented with zinc and zinc nanoparticles. Aquac. Rep..

[41] Suttle NF (2022). Mineral Nutrition of Livestock.

[42] Cui H (2018). Effects of different sources and levels of zinc on growth performance, nutrient digestibility, and fur quality of growing-furring male mink (Mustela Vison). Biol. Trace Elem. Res..

[43] Fawaz MA, Südekum KH, Hassan HA, Abdel-Wareth AAA (2019). Effects of nanoparticles of zinc oxide on productive performance of laying hens–a review. SVU-Int. J. Agric. Sci..

[44] Bortoluzzi, C. *et al.* Zinc source modulates intestinal inflammation and intestinal integrity of broiler chickens challenged with coccidia and Clostridium perfringens. *Poult. Sci.* **98**(5), 2211–2219. 10.3382/ps/pey587 (2019). 10.3382/ps/pey587 30668786

[45] Falchi L, Khalil WA, Hassan M, Marei WFA (2018). Perspectives of nanotechnology in male fertility and sperm function. Int. J. Vet. Sci. Med..

[46] Romeo A, Vacchina V, Legros S, Doelsch E (2014). Zinc fate in animal husbandry systems. Metallomics.

[47] Patil SS, Kore KB, Kumar P (2009). Nanotechnology and its applications in veterinary and animal science. Vet. World.

[48] Ganaie AH (2013). Biochemical and physiological changes during thermal stress in bovines: A review. Iran. J. Appl. Anim. Sci..

[49] Rajendran, D., Thulasi, A., Jash, S., Selvaraju, S. & Rao, S. B. N. Synthesis and application of nano minerals in livestock industry. In *Animal nutrition and reproductive physiology (recent concepts).* Satish Ser. Publ. House, Delhi 517–530 (2013).

[50] Moce, E., Arouca, M., Lavara, R. & Pascual, J. J. Effect of dietary zinc and vitamin supplementation on semen characteristics of high growth rate males during summer season. in *Proceedings of the 7th World Rabbit Congress* 203–209 (2000).

[51] El-Masry, K. A., Nasr, A. S. & Kamal, T. H. Influences of season and dietary supplementation with selenium and vitamin E or zinc on some blood constituents and semen quality of New Zealand White rabbit males. *World Rabbit Sci.* **2**(3), 79–86 (1994).

[52] Raeeszadeh M, Shokrollahi B, Akbari A (2022). Superior effect of broccoli methanolic extract on control of oxidative damage of sperm cryopreservation and reproductive performance in rats: A comparison with vitamin C and E antioxidant. Theriogenology.

[53] Zhang C (2015). Effect of different Nano-zinc levels in dietary on semen quality, activities of antioxidant enzyme and expression of copper zinc superoxide in epididymis of ram lambs. Sci. Agric. Sin..

[54] Jahanbin R (2015). Effect of zinc nano-complex on bull semen quality after freeze-thawing process. Anim. Prod..

[55] Afifi, M., Almaghrabi, O. A. & Kadasa, N. M. Ameliorative effect of zinc oxide nanoparticles on antioxidants and sperm characteristics in streptozotocin-induced diabetic rat testes. *Biomed. Res. Int.* **2015**, 153573. 10.1155/2015/153573 (2015). 10.1155/2015/153573 PMC4637006 26581756

[56] El-Gindy YM (2022). Improvement in quality and storage ability of rabbit semen by using black or thyme seed as dietary supplementation. J. Anim. Physiol. Anim. Nutr. (Berl).

[57] Geary TW (2016). Effect of supplemental trace mineral level and form on peripubertal bulls. Anim. Reprod. Sci..

[58] Kumar N, Verma RP, Singh LP, Varshney VP, Dass RS (2006). Effect of different levels and sources of zinc supplementation on quantitative and qualitative semen attributes and serum testosterone level in crossbred cattle (Bos indicus $\bf\times $ Bos taurus) bulls. Reprod. Nutr. Dev..

[59] Salim HM, Jo C, Lee BD (2008). Zinc in broiler feeding and nutrition. Avian Biol. Res..

[60] Kowsar R, Ronasi S, Sadeghi N, Sadeghi K, Miyamoto A (2021). Epidermal growth factor alleviates the negative impact of urea on frozen-thawed bovine sperm, but the subsequent developmental competence is compromised. Sci. Rep..

[61] Abaspour Aporvari MH, Mamoei M, Tabatabaei Vakili S, Zareei M, Dadashpour Davachi N (2018). The effect of oral administration of zinc oxide nanoparticles on quantitative and qualitative properties of arabic ram sperm and some antioxidant parameters of seminal plasma in the non-breeding season. Arch. Razi Inst..

[62] Wong WY (2002). Effects of folic acid and zinc sulfate on male factor subfertility: A double-blind, randomized, placebo-controlled trial. Fertil. Steril..

[63] Ghorbani A, Moeini MM, Souri M, Hajarian H (2018). Influences of dietary selenium, zinc and their combination on semen characteristics and testosterone concentration in mature rams during breeding season. J. Appl. Anim. Res..

[64] Dhama K (2014). Growth promoters and novel feed additives improving poultry production and health, bioactive principles and beneficial applications: The trends and advances-a review. Int. J. Pharmacol..

[65] Kumar P, Yadav B, Yadav S (2013). Effect of zinc and selenium supplementation on antioxidative status of seminal plasma and testosterone, T4 and T3 level in goat blood serum. J. Appl. Anim. Res..

[66] Ehala-Aleksejev K, Punab M (2016). Serum hepatic enzyme activity in relation to semen quality and serum reproductive hormone levels among Estonian fertile Men. Andrology.

[67] Al-Sagheer, A. A., Abdel-Rahman, G., Ayyat, M. S., Gabr, H. A. & Elsisi, G. F. Productive performance response of growing rabbits to dietary protein reduction and supplementation of pyridoxine, protease, and zinc. *An. Acad. Bras. Cienc.* **92**(3), 10.1590/0001-3765202020180989 (2020). 10.1590/0001-3765202020180989 32876141

[68] Abdel-Wareth AAA, Amer SA, Mobashar M, El-Sayed HGM (2022). Use of zinc oxide nanoparticles in the growing rabbit diets to mitigate hot environmental conditions for sustainable production and improved meat quality. BMC Vet. Res..

[69] El-Moghazy M, El-Fadaly HA, Khalifa EI, Mohamed MA (2019). Effect of dietary zinc-methionine on growth, carcass traits, antioxidants and immunity of growing rabbits. J. Anim. Poult. Prod..

[70] El-Hamid A, El-Speiy M, Hassan S, Habbibe M (2018). Performance, immunity response, blood biochemical and hematological traits of growing male rabbits affected by type water with zinc. Egypt. J. Rabbit Sci..

[71] Raslan MAH, Ismail ZSH, Abdel-Wareth AAA (2020). Application of zinc oxide nanoparticles on productive performance in rabbit nutrition: A review. SVU-Int. J. Agric. Sci..

[72] El-Gindy, Y. M. *et al.* Enhancing semen quality, antioxidant status and sex hormones of V-line rabbit bucks fed on supplemented diets with dried moringa leaves. *Anim. Biotechnol.* 1–10. 10.1080/10495398.2022.2110109 (2022). 10.1080/10495398.2022.2110109 36000985

[73] Prasad AS (2009). Zinc: Role in immunity, oxidative stress and chronic inflammation. Curr. Opin. Clin. Nutr. Metab. Care.

[74] Alissa EM, Bahijri SM, Lamb DJ, Ferns GAA (2004). The effects of coadministration of dietary copper and zinc supplements on atherosclerosis, antioxidant enzymes and indices of lipid peroxidation in the cholesterol-fed rabbit. Int. J. Exp. Pathol..

[75] Hau J, Schapiro SJ, Van Hoosier Jr GL (2002). Handbook of Laboratory Animal Science: Animal Models.

[76] El-Gindy, Y. M., Zahran, S. M., Hassan, M. A. & Sabir, S. A. Effect on physiological parameters and semen quality upon oral administration of fresh onion juice to V-line rabbit buck during severe heat stress. *Anim. Biotechnol.* 1–9. 10.1080/10495398.2022.207018 (2022). 10.1080/10495398.2022.2070184 35544609

[77] Arif M (2019). Impacts of supplementing broiler diets with a powder mixture of black cumin, Moringa and chicory seeds. S. Afr. J. Anim. Sci..

